# Comprehensive energy efficiency optimization algorithm for steel load considering network reconstruction and demand response

**DOI:** 10.1038/s41598-023-46804-7

**Published:** 2023-11-21

**Authors:** Yuxiu Zang, Shunjiang Wang, Weichun Ge, Yaping Li, Jia Cui

**Affiliations:** 1 School of electrical engineering, Shenyang University of Technology, No. 111, Shenliao West Road, Economic & Technological Development Zone, Shenyang, 110000 China; 2 State Grid Shenyang Electric Power Supply Company, No. 94, Bajing Street, Heping District, Shenyang, 110000 China; 3 State Grid Liaoning Electric Power Supply CO., LTD., No.18, Ningbo Road, Heping District, Shenyang, 110000 China; 4 China Electric Power Research Institute Co., Ltd, No. 8, Nanrui Road, Gulou District, Nanjing, 210000 China

**Keywords:** Electrical and electronic engineering, Energy grids and networks, Power distribution, Power stations

## Abstract

Industrial loads are usually energy intensive and inefficient. The optimization of energy efficiency management in steel plants is still in the early stage of development. Considering the topology of power grid, it is an urgent problem to improve the operation economy and load side energy efficiency of steel plants. In this paper, a two-level collaborative optimization method is proposed, which takes into account the dynamic reconstruction cost, transmission loss cost, energy cost and demand response benefit. The upper level objective is the optimization of topology in the grid structure to optimize the power loss and dynamic reconstruction costs of the grid. The lower level is the energy cost considering demand response, real time price and dynamic demand response price. Firstly, the mathematical models of stable load, impact load and the steel production line load are built. The key parameters are identified by the Back Propagation neural network algorithm according to the actual production data. Secondly, considering the constraints of grid structure and load operation capacity, the impact of dynamic grid loss and real-time dynamic electricity price on the energy efficiency of the whole grid are analyzed in depth. The optimal operation model considering the dynamic reconfiguration and grid tramission loss of distribution network is built. Taking a steel plant park in Northeast China as an example, it is proved that the optimization model can improve energy efficiency on the load side by optimizing energy consumption and demand response participation time on load side. The energy cost is reduced by 17.77% on the load side, the network loss is reduced by 1.8%, and the operating cost of the power grid is reduced by 26.2%, which has a positive effect on improving energy utilization efficiency, reducing distribution network loss, and improving overall economic efficiency.

## Introduction

At present, the energy consumption of industrial load is large, energy efficiency is low, the load side energy efficiency management is relatively weak. At the same time, the existing grid structure and the power consumption behavior of the load side are not be planned and made full use, resulting in the uneven distribution of power flow, high power loss rate and operating cost, which is a subject that needs to be studied. In terms of modeling steel loads, it divides steel loads into sustained impact loads, intermittent impact loads, and stable loads. By analyzing the relevant energy consumption behavior of these three types of load loads, The authors establish time-domain models of power characteristics for different types of loads in Ref.^1^. In Ref.^2^, an evaluation model is proposed to quantify the provisions of flexibility of Ladle furnaces (LFs) as cuttable loads. The regulation capacity of LFs is evaluated and the electricity costs before and after power adjustments are compared. Three adjustable production processes are considered interruptible, transferrable, and reducible ones^3^. By analyzing the power characteristics of steel plant based on the above references, the fitting error with data is relatively large and the description of power characteristics are not accurate. When studying the load energy efficiency and power grid reconstruction, it is not only necessary to consider the demand response characteristics model of steel loads^4^, but also their production characteristics. Considering the comprehensive energy efficiency of load energy consumption costs and demand response^5^ benefits in the steel plant park, it is necessary to improve the universality of the load side model and the accuracy of power fitting.

In terms of reducing energy consumption^6^ in steel plant with the consideration of real time price^7^, the author studies the adjustable operating speed of continuous casting process equipment to further reduce energy consumption in Ref.^8^. In Ref.^9^, author proposes a real-time decision-making model for load management in industrial production processes facing constantly changing real-time prices. In Ref.^10^, the author abstracts the resource and time constraints existing in the steel production process into a mathematical model, and establishes a two-level optimal day ahead model that comprehensively considers power grid scheduling and enterprise consumer interests.

In terms of power grid^11^ reconstruction, A multi-objective dynamic reconfiguration is modeled based on the time-varying load distribution network considering network active power loss, static voltage stability, and load balance in Ref.^12^. In Ref.^13^, the author improves the Grey Wolf optimization algorithm by adding three strategies to the algorithm, comprehensively improving its performance and applying it to the distribution network reconstruction problem to obtain the optimal solution. A distribution network reconstruction technology based on an improved selective binary particle swarm optimization algorithm is proposed in the^14^, which improves the iterative performance of the algorithm and promotes better exploration of the search space.

However, The current references only focus on the arrangement of the distribution network, the arrangment of the power side or both of them. The operation optimization management of the load side is failure to consider collaborative optimization with the distribution network side. The dynamic reconfiguration technology needs to be fully utilized, the collaborative optimization between load side and distribution network side should be considered. Only in this way can the comprehensive cost of distribution network and load be reduced comprehensively, and the operation of the distribution network be optimized.

### The motivations and contributions

The model built should have good fitting and universality for subsequent research. When optimizing, benefit should be increased and the total electricity cost of load should be reduced by participating in demand response without affecting production. The power consumption on the load side will affect the power flow of the power grid. By adjusting the topology structure of the power grid appropriately, static network loss can be reduced and the power flow of the power grid can be optimized. The contributions of the paper are as follows

Firstly, the models should be built with good fitting and universality. The loads are devided into three types in the paper: stable load, impact load, and production line load based on the electrical properties and time-domain characteristics of the energy side load. Secondly, a two-level collaborative optimization method is proposed that considers the cost of static and dynamic power grid reconstruction and load energy efficiency. A load energy efficiency optimization model is proposed based on the definition of energy efficiency ratio, which considers the load side energy efficiency optimization model with real time electricity price and demand response optimization strategy with dynamic demand response electricity price. Thirdly, by further calculating the cost of static network loss and dynamic network reconstruction, the energy efficiency optimization on the power grid and load side can be improved, and the economy of energy transmission and energy consume can be improved.The relationship between distribution network cost and load economic management is mutually constrained and influenced. The study is mainly divided into the following contents:

1. Firstly, the stable load, impact load and production line load models of steel plant load are built, and the key parameters are identified by using BP neural network algorithm based on historical data. By analyzing the error of model fitting through the large amount of data, the results show that the fitting results meet the practical requirements of engineering and have a certain degree of universality.The stable load models are mainly divided into two categories: Load with high power and stable feature, and Load with low power and fluctuating feature. The fitting error maximum value are 4.5% and 6%, respectively. It is a optimization problem concerning the steel plant load production planning and comprehensive cost that considers energy costs and demand response. A load energy efficiency optimization model based on real time price and dynamic demand response price is built. Secondly, taking dynamic reconstruction cost and static network loss as optimization objectives, the distribution network topology is optimized and reconstructed in time segments. It avoids the mechanical loss caused by frequent operation, improves the low voltage problem caused by line overload, and improves the power loss of the line.
2. The data of an industrial park in Northeast China is adopted in the paper for simulation. The load energy efficiency optimization is calculated, and the efficiency improvement ability of the optimization model is verified. The results show that the error rate of the stable load model is 6%, the average error of the shock load is 8%, the loss of the distribution network is reduced by 1.8%, and the operating cost is saved by 17.77%. By improving the load side energy consume planning and demand response, the energy cost of steel plant load can be reduced directly, the overload of lines can be reduced indirectly, and the line loss can be directly reduced through dynamic reconfiguration of lines.

### The mathematical model of industrial loads

Industrial loads can be divided into three main types of loads. The static load is with relatively stable power characteristics, the impact load of is with high-frequency harmonics, showing "banded" power characteristics, and the production line load is composed of a variety of equipments.

A ZIP model is used to represent the static model:

1 $$\begin{aligned} & P^{\rm I} = P_{0} \left[ {A_{p} \left( {\frac{U}{{U_{0} }}} \right)^{2} + B_{p} \left( {\frac{U}{{U_{0} }}} \right) + C_{p} } \right] \\ & A_{p} + B_{p} + C_{p} = 1 \\ \end{aligned}$$

$A_{p}$, $B_{p}$, $C_{p}$ are stable load coefficients, $U_{0}$ is the steady-state voltage of the load, $U$ is the actual voltage, and $P_{0}$ is the steady-state power.

When the impact load itself requires a large power, the system will have a power shock. It is the difference between the static load and impact load, namely "initiative". The power absorbed from the system is determined by its own production characteristics. The shock model can be expressed as follows:

2 $$\left\{ \begin{gathered} \frac{{{\text{d}}E_{q}^{\prime } }}{{{\text{d}}t}} = - \frac{1}{{T_{{{\text{d}}0}} }}\left[ {E_{q}^{\prime } - \left( {X - X^{\prime } } \right)i_{d} } \right] + \omega_{0} \left( {\omega - 1} \right)E_{d}^{\prime } \hfill \\ \frac{{{\text{d}}E_{d}^{\prime } }}{{{\text{d}}t}} = - \frac{1}{{T_{{{\text{d}}0}} }}\left[ {E_{d}^{\prime } + \left( {X - X^{\prime } } \right)i_{q} } \right] + \omega_{0} \left( {\omega - 1} \right)E_{q}^{\prime } \hfill \\ \frac{d\omega }{{dt}} = - \frac{1}{2H}\left( {T_{m} - T_{e} } \right) \hfill \\ {{{\text{d}}\delta } \mathord{\left/ {\vphantom {{{\text{d}}\delta } {{\text{d}}t = \left( {\omega - 1} \right)\omega_{0} }}} \right. \kern-0pt} {{\text{d}}t = \left( {\omega - 1} \right)\omega_{0} }} \hfill \\ \end{gathered} \right.$$

3 $$\begin{aligned} i_{d} & = \frac{1}{{R_{s}^{2} + X^{\prime 2} }}\left[ {R_{s} \left( {U_{d} - E_{d}^{\prime } } \right) + X^{\prime } \left( {U_{q} - E_{q}^{\prime } } \right)} \right] \\ i_{q} & = \frac{1}{{R_{s}^{2} + X^{\prime 2} }}\left[ {R_{s} \left( {U_{q} - E_{q}^{\prime } } \right) + X^{\prime } \left( {U_{d} - E_{d}^{\prime } } \right)} \right] \\ \end{aligned}$$

4 $$\begin{aligned} P_{{{\text{r1}}}}^{{{\rm I}{\rm I}}} & = U_{d} i_{d} + U_{q} i_{q} \\ Q_{{{\text{r1}}}}^{{{\rm I}{\rm I}}} & = U_{q} i_{d} - U_{d} i_{q} \\ \end{aligned}$$

$E_{d}^{\prime}$, $E_{q}^{\prime}$ are respectively the $d$, $q$ components of the transient electromotive force of the induction motor. $i_{d}$, $i_{q}$ are respectively the $d$, $q$ components of the asynchronous motor current; $U_{d}$, $U_{q}$ are respectively the $d$, $q$ components of the asynchronous motor voltage. $\omega$ is the angular velocity of rotation. $\delta$ is the Angle between the $q$ axis and the $x$ axis. $P_{r1}$ is the load active power; $Q_{{{\text{r1}}}}$ is the reactive power of air conditioning load. $\omega_{0}$ is the electric angular velocity. $T_{{{\text{d}}0}} = \left( {X_{{\text{r}}} + X_{{\text{m}}} } \right)/(\omega_{0} * R_{{\text{r}}} )$ is the loop time constant of $d$ axis rotor in open circuit. $R_{{\text{r}}}$ is the rotor resistance. $R_{s}$ is the stator resistance. $H$ is the rotor inertia time constant. $T_{{\text{m}}} = T_{{{\text{m0}}}} (A\omega^{2} + B\omega + C + D\omega^{E} )$ is the mechanical torque of asynchronous motor; $T_{{{\text{m0}}}}$ is the load rate, $A,B,C,D$ is the constant. $T_{e}$ is the electromagnetic torque of asynchronous motor. $X = X_{r} + X_{m}$, $X^{\prime } = X_{s} + X_{m} //X_{r}$. Where $X,X^{\prime } ,X_{r} ,X_{m} ,X_{s}$ are respectively open rotor reactance, rotor blocking reactance, rotor reactance, fixed rotor mutual reactance and stator reactance.

5 $$\begin{aligned} & P_{r1} \left( t \right) = \sum\limits_{i = 1}^{{n_{r} }} {P_{r1,i}^{{{\rm I}{\rm I}}} \left( t \right)} = P_{r1,1}^{{{\rm I}{\rm I}}} \left( t \right) + P_{r1,2}^{{{\rm I}{\rm I}}} \left( t \right) + \cdots + P_{{r1,n_{r} }}^{{{\rm I}{\rm I}}} \left( t \right) \\ & \quad = P_{r1,1}^{{{\rm I}{\rm I}}} \left( t \right) + P_{r1,1}^{{{\rm I}{\rm I}}} \left( {t - \Delta T_{2} } \right) + \cdots + P_{r1,1}^{{{\rm I}{\rm I}}} \left( {t - \Delta T_{{n_{r} }} } \right) \\ \end{aligned}$$

6 $$\begin{aligned} & P_{f1} \left( t \right) = \sum\limits_{j = 1}^{{n_{f} }} {P_{f1,j}^{{{\rm I}{\rm I}}} \left( t \right)} = P_{f1,1}^{{{\rm I}{\rm I}}} \left( t \right) + P_{f1,2}^{{{\rm I}{\rm I}}} \left( t \right) + \cdots + P_{{f1,n_{f} }}^{{{\rm I}{\rm I}}} \left( t \right) \\ & \quad = P_{f1,1}^{{{\rm I}{\rm I}}} \left( t \right) + P_{f1,1}^{{{\rm I}{\rm I}}} \left( {t - \Delta T_{2} } \right) + \cdots + P_{f1,1}^{{{\rm I}{\rm I}}} \left( {t - \Delta T_{{n_{f} }} } \right) \\ \end{aligned}$$

The number of billet $n_{s}$, the number of roughing mills $n_{r}$, and the number of finishing mills $n_{f}$ are considered. Each billet passes through the roughing mills of $n_{r}$ and then finishing mills of $n_{f}$ before forming a steel sheet. The power generated in the whole rolling process is described. For the $i$ roughing mill, the power of rolling the first billet can be expressed by Eq. (2):

The total power of steel rolling production line can be expressed as:

7 $$P^{{{\rm I}{\rm I}{\rm I}}} \left( t \right) = \sum\limits_{{r = r_{1} }}^{R} {P_{r1} \left( t \right)} + \sum\limits_{{f = f_{1} }}^{F} {P_{f1} \left( t \right)}$$

The steel rolling process has strict production process flow, it needs to start and stop in sequence, subject to the coupling restriction of rolling sequence as follows:

8 $$\alpha_{b,t} \le \frac{1}{T}\sum\limits_{k = 1}^{t - 1} {\alpha_{c,k} }$$

Load $b$ can only run after load $c$ is finished, that is, the pre-load constraint is:

9 $$\alpha_{b,t} - \alpha_{b,t - 1} \le \sum\limits_{k = 1}^{t - 1} {\alpha_{b,t} }$$

The total load at time $t$ can be expressed as:

where, $\partial_{u}^{\rm I}$, $\partial_{r}^{{{\rm I}{\rm I}}}$, $\partial_{c}^{{{\rm I}{\rm I}{\rm I}}}$ are state variables of static load, impact load and production line load. $U$,$R$, $C$ are three kinds of load quantity respectively. $P_{u,k}^{\rm I}$ is the power of the u static load at time k. $P_{r,k}^{{{\rm I}{\rm I}}}$ is the r shock load power at time k. $P_{c,k}^{{{\rm I}{\rm I}{\rm I}}}$ respectively refers to the load power of the c production line at time *k*.

The BP algorithm calculates errors in backpropagation, effectively reducing training time. Therefore,the BP algorithm has high learning efficiency. The error of the algorithm gradually decreases and will eventually converge to the minimum error. Therefore, the BP algorithm has certain advantages in parameter identification^15^ in the paper. the BP neural network algorithm^16–18^ is as shown in equation (10)–(13).

10 $$net_{\tau } = \sum\limits_{s = 1}^{S} {w_{\tau s} } u_{s} + a_{\tau }$$

11 $$\sigma_{\tau } = \phi \left( {net_{\tau } } \right) = \phi \left( {\sum\limits_{\tau = 1}^{\omega } {w_{\tau s} x_{s} + a_{s} } } \right)$$

12 $$net_{s} = \sum\limits_{\tau = 1}^{\varpi } {w_{\tau s} } \phi \left( {\sum\limits_{\tau = 1}^{S} {w_{\tau s} } u_{s} + a_{s} } \right) + \beta_{s}$$

13 $$\sigma_{k} = \psi \left( {net_{s} } \right) = \psi \left[ {\sum\limits_{\tau = 1}^{\varpi } {w_{\tau s} } \phi \left( {\sum\limits_{\tau = 1}^{S} {w_{\tau s} } u_{s} + a_{s} } \right) + \beta_{s} } \right]$$

**Figure 1:**
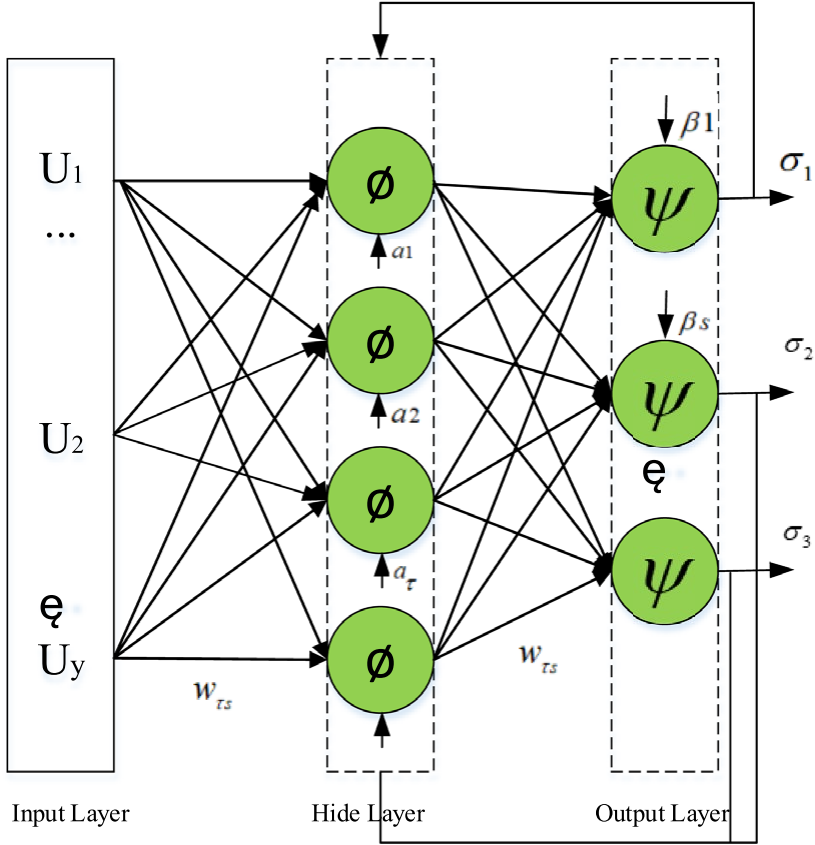
Structure diagram of three-layer neural network. (Identification of key parameters based on neural network^9–11^.Through the load cluster analysis of the adjustable capacity of the power load, the load power consumption data, the factors affecting the power consumption, and the adjustable time. For the loads of the same class after clustering, the BP neural network is used to learn and predict the load power. In figure, $U_{s}$ represents the input of the node s in the input layer, $w_{\tau Y}$ represents the weight between the node $\tau$ in the hidden layer and the node Y in the input layer, $a_{\varpi }$ is the threshold of the node $\varpi$ in the hidden layer, φ is the activation function of the hidden layer, $w_{\tau F}$ represents the weight between the node $\tau$ in the output layer and the node F in the hidden layer, $\beta_{s}$ is the threshold of the node s in the output layer, Y is the dimension of the input signal, $\varpi$ is the total number of hidden layer nodes, F is the node dimension in the output layer, ψ is the excitation function of the output layer, $\sigma_{k}$ represents the output of the node k. The calculation of the neural network is mainly divided into two steps, the forward calculation of the data is performed, and the reverse correction of the weights and thresholds of each layer is performed.)

In Fig. 1, $U_{s}$ represents the input of the node s in the input layer, $w_{\tau Y}$ represents the weight between the node $\tau$ in the hidden layer and the node Y in the input layer, $a_{\varpi }$ is the threshold of the node $\varpi$ in the hidden layer, φ is the activation function of the hidden layer, $w_{\tau F}$ represents the weight between the node $\tau$ in the output layer and the node $F$ in the hidden layer, $\beta_{s}$ is the threshold of the node $s$ in the output layer, $Y$ is the dimension of the input signal, $\varpi$ is the total number of hidden layer nodes, $F$ is the node dimension in the output layer, ψ is the excitation function of the output layer, $\sigma_{k}$ represents the output of the node $k$. The calculation of the neural network is mainly divided into two steps, the forward calculation of the data is performed, and the reverse correction of the weights and thresholds of each layer is performed.

Backpropagation of the error signal:

14 $$\Delta w_{s\tau } = \eta \sum\limits_{p = 1}^{P} {\sum\limits_{s = 1}^{S} {\left( {T_{s}^{P} - \sigma_{s}^{P} } \right)} } \psi^{\prime } \left( {net_{s} } \right)\sigma_{\tau }$$

15 $$\Delta \alpha_{k} = \eta \sum\limits_{p = 1}^{P} {\sum\limits_{s = 1}^{S} {\left( {T_{s}^{P} - \sigma_{s}^{P} } \right)} } \psi^{\prime } \left( {net_{s} } \right)$$

16 $$\Delta w_{ij} = \eta \sum\limits_{p = 1}^{P} {\sum\limits_{s = 1}^{S} {\left( {T_{s}^{P} - \sigma_{s}^{P} } \right)} } \psi^{\prime } \left( {net_{s} } \right)w_{s\tau } \phi^{\prime } \left( {net_{\tau } } \right)u_{s}$$

17 $$\Delta a_{\tau } = \Delta w_{ij} = \eta \sum\limits_{p = 1}^{P} {\sum\limits_{s = 1}^{S} {\left( {T_{s}^{P} - \sigma_{s}^{P} } \right)} } \psi^{\prime } \left( {net_{s} } \right)w_{s\tau } \phi^{\prime } \left( {net_{\tau } } \right)$$

$\Delta w_{s\tau }$ is the weight correction amount between the node s in the hidden layer and the node $\tau$ in the output layer, $\Delta \beta_{s}$ is the threshold correction amount of the node s of the output layer. $\Delta w_{\tau s}$ is the weight correction amount from the node i in the input layer to the node j in the hidden layer, $\Delta a_{\tau }$ is the threshold correction of the node i in the hidden layer, $p$ is the load sample index. $\eta$ is the learning rate, and P is the total number of training samples. In this paper, the input of the input layer is power, voltage and impedance, and the output of the output layer is the power model parameters $A_{p}$, $B_{p}$, $C_{p}$,$\alpha ,\beta$.

### Load energy efficiency optimization model considering Network Reconstruction

Load energy efficiency is the benefit generated by energy consumption in the production process, is a kind of input–output ratio, refers to the ratio between energy costs and product benefits. The cost model of electricity in the running period $T$ is as follows:

18 $$P_{\sum } \left( t \right) = \sum\limits_{u = 1}^{U} {\partial_{u}^{\rm I} P_{u}^{\rm I} \left( t \right)} + \sum\limits_{r = 1}^{R} {\partial_{r}^{{{\rm I}{\rm I}}} P_{r}^{{{\rm I}{\rm I}}} \left( t \right)} + \sum\limits_{c = 1}^{C} {\partial_{c}^{{{\rm I}{\rm I}{\rm I}}} P_{c}^{{{\rm I}{\rm I}{\rm I}}} \left( t \right)}$$

19 $$C_{1} = \sum\limits_{t = 1}^{T} {\sum\limits_{u = 1}^{U} {\sum\limits_{r = 1}^{R} {\sum\limits_{s = 1}^{S} {\left( {C_{u}^{\rm I} \left( t \right) + C_{r}^{{{\rm I}{\rm I}}} \left( t \right) + C_{s}^{{{\rm I}{\rm I}{\rm I}}} \left( t \right)} \right)} } } }$$

$$\begin{aligned} C_{u\sum }^{\rm I} & = \sum\limits_{t = 1}^{T} {\sum\limits_{u = 1}^{U} {\partial_{u}^{\rm I} P_{u}^{\rm I} \left( t \right)} c_{t} } \\ C_{r\sum }^{{{\rm I}{\rm I}}} & = \sum\limits_{t = 1}^{T} {\sum\limits_{r = 1}^{R} {\partial_{r}^{{{\rm I}{\rm I}}} P_{r}^{{{\rm I}{\rm I}}} \left( t \right)c_{t} } } \\ C_{s\sum }^{{{\rm I}{\rm I}{\rm I}}} & = \sum\limits_{t = 1}^{T} {\sum\limits_{s = 1}^{S} {\partial_{s}^{{{\rm I}{\rm I}{\rm I}}} P_{s}^{{{\rm I}{\rm I}{\rm I}}} \left( t \right)c_{t} } } \\ \end{aligned}$$

where $C_{u}^{\rm I}$, $C_{r}^{{{\rm I}{\rm I}}}$, $C_{s}^{{{\rm I}{\rm I}{\rm I}}}$ respectively refers to the electricity cost of static load, impact load and production line load within a scheduling cycle $T$.$c_{t}$ is as the electric price time $t$, $C_{1}$ is the total cost of load electricity.

The benefit cost of load participation demand network demand response^19,20^ is:

20 $$F = \sum\limits_{t = 1}^{T} {\sum\limits_{u = 1}^{U} {\sum\limits_{r = 1}^{R} {\sum\limits_{s = 1}^{S} {\left( {C_{u}^{{\prime {\rm I}}} \left( t \right) + C_{r}^{{\prime {\rm I}{\rm I}}} \left( t \right) + C_{s}^{{\prime {\rm I}{\rm I}{\rm I}}} \left( t \right)} \right)} } } }$$

21 $$\begin{aligned} C_{u\sum }^{{\prime {\rm I}}} & = \sum\limits_{t = 1}^{T} {\sum\limits_{u = 1}^{U} {\partial_{u}^{\rm I} \Delta P_{u}^{\rm I} \left( t \right)} c_{t}^{\prime } } \\ C_{r\sum }^{{\prime {\rm I}{\rm I}}} & = \sum\limits_{t = 1}^{T} {\sum\limits_{r = 1}^{R} {\partial_{r}^{{{\rm I}{\rm I}}} \Delta P_{r}^{{{\rm I}{\rm I}}} \left( t \right)c_{t}^{\prime } } } \\ C_{s\sum }^{{\prime {\rm I}{\rm I}{\rm I}}} & = \sum\limits_{t = 1}^{T} {\sum\limits_{s = 1}^{S} {\partial_{s}^{{{\rm I}{\rm I}{\rm I}}} \Delta P_{s}^{{{\rm I}{\rm I}{\rm I}}} \left( t \right)c_{t}^{\prime } } } \\ \end{aligned}$$

where, $C_{u}^{{\prime {\rm I}}} \left( t \right)$,$C_{r}^{{\prime {\rm I}{\rm I}}} \left( t \right)$,$C_{s}^{{\prime {\rm I}{\rm I}{\rm I}}} \left( t \right)$ respectively represent the static load, impact load and production line load participating in the grid demand response within a dispatching cycle $t$. $c_{t}^{\prime }$ is the electricity price at time $t$. $F$ is the total revenue of load demand response revenue.

To sum up, the output value model of a single type of load in the running period $K$ is expressed as follows:

22 $$E_{k}^{{}} = \sum\limits_{k = 1}^{K} {{{W_{k} \cdot x} \mathord{\left/ {\vphantom {{W_{k} \cdot x} \eta }} \right. \kern-0pt} \eta }_{k} }$$

$W_{k}$ is the total qualified product quantity. $x$ is the product price. $\eta_{k}$ is the success rate of pipeline production.

The optimal energy consumption model of load can be expressed as follows:

23 $$\min \eta = {{\left( {C_{1} - F} \right)} \mathord{\left/ {\vphantom {{\left( {C_{1} - F} \right)} {E_{{\text{k}}} }}} \right. \kern-0pt} {E_{{\text{k}}} }}$$

The loss of the grid can be divided into two parts: the static loss and the dynamic loss. Static loss refers to the network loss based on the current power flow when the network topology is unchanged. Dynamic loss is caused by the change of the running state of switches in the distribution network^21–23^. The Active management means can actively adjust the grid topology and power flow of distribution network^24^. The combination of the two can greatly reduce the network loss and improve the economy of the distribution network operation.

The cost of grid side operation mainly includes reconstruction cost $C_{2}$, operation cost $C_{3}$ and power flow network loss $C_{4}$.

24 $$C = C_{2} + C_{3} + C_{4} = \sum\limits_{n = 1}^{N} {\left| {k_{n}^{1} - k_{n}^{0} } \right|} + mP_{l} + \sum\limits_{n = 1}^{N} {k_{n} r_{n} \left( {P_{n}^{2} + Q_{n}^{2} } \right)} /U_{n}^{2}$$

where, $m$ is the operation and maintenance cost factor of the line, which is generally 1% to 2%. $P_{l}$ is the capacity of the. line $l$ in the line set. $n$ is the number of the branch. $N$. $r_{n}$, $P_{n}$, $Q_{n}$, $U_{n}^{{}}$ are the impedance, active power, reactive power and terminal voltage of the branch. $k_{n}$ represents tthe switch state of the branch, variable 0–1. 0 represents the branch switch is off, 1 represents the switch is on. $F$ is the number of switch actions, $k_{n}^{1}$ and $k_{n}^{0}$ are the switch status of branch $n$ before and after reconstruction.

Upper and lower limits on transmission capacity of distribution lines:

25 $$P_{g,d\min } \le P_{g,d} \le P_{g,d\max }$$

where, $P_{g,d}$ is the actual transmission capacity of line $g$. $P_{g,d\min }$, $P_{g,d\max }$ are the minimum and maximum transmission capacities of line $g$.

26 $$P_{h,z} - P_{h,f} = \sum\limits_{g = 1}^{H} {B_{hm} } \left( {\theta_{h} - \theta_{m} } \right)$$

$P_{h,z}$,$P_{h,f}$ are the active power and load demand injected by node $h$. $B_{hm}$ is the susceptance value of the line between $h,m$. $H$ is the node set of the transmission line, $h,m \in H$.

The phase Angle constraint:

27 $$\theta_{h,\min } \le \theta_{h} \le \theta_{h,\max }$$

For any node $h$ in the distribution network, $\theta_{h,\min }$ and $\theta_{h,\max }$ are the upper and lower limits of phase Angle $h$ of node.

Voltage constraints Constraints on grid operation

28 $$U_{j\min } \le U_{j} \le U_{j\max }$$

Restriction of consumer adjustment ability:

29 $$P_{n\min } \le P_{n} \le P_{n\max }$$

$E$ is the optimal energy efficiency of the whole network. $a_{1} ,a_{2} ,...,a_{n}$ is the optimal profit conversion per power of the load $N$. $P_{1} ,P_{2} ,...,P_{n}$ is the operating power of the load $N$. $P_{s}$ indicates network loss. $A\left( t \right)$ is the dynamic electric price. $P_{l\min }$, $P_{n\min }$ are the lower limits of line power distribution and consumer energy capacity respectively. $P_{l\max }$, $P_{n\max }$ are the upper limits of line power distribution and consumer energy capacity respectively.

30 $$\left\{ \begin{gathered} P_{i} = U_{i} \sum\limits_{j \in i} {U_{j} \left( {G_{ij} \cos \theta_{ij} + B_{ij} \sin \theta_{ij} } \right)} \hfill \\ Q_{i} = U_{i} \sum\limits_{j \in i} {U_{j} \left( {G_{ij} \sin \theta_{ij} + B_{ij} \cos \theta_{ij} } \right)} \hfill \\ \end{gathered} \right.$$

where, $P_{i}$, $Q_{i}$ respectively represent the equivalent active and reactive power injection of node $i$. $U_{i}$,$U_{j}$ respectively represent the voltage of nodes $i$ and $j$. $G_{ij}$ $B_{ij}$ respectively represent the real and imaginary parts of the node admittance matrix. $\theta_{ij}$ is the phase difference between node $i,j$.

### The two-level optimization model and algorithm solution

The form of the two-level^25^ optimization model is as follows:

31 $$\begin{gathered} \left\{ \begin{gathered} \min C = \sum\limits_{n = 1}^{N} {\left| {k_{n}^{1} - k_{n}^{0} } \right|} + mP_{l} + \sum\limits_{n = 1}^{N} {k_{n} r_{n} \left( {P_{n}^{2} + Q_{n}^{2} } \right)} /U_{n}^{2} \hfill \\ {\text{s}}{\text{.t}}{.}\;(26) \sim (31) \hfill \\ \end{gathered} \right. \hfill \\ \left\{ \begin{gathered} \min \;\eta = {{c_{1} } \mathord{\left/ {\vphantom {{c_{1} } {E_{{\text{k}}} }}} \right. \kern-0pt} {E_{{\text{k}}} }} \hfill \\ {\text{s}}{\text{.t}}{.}\;(8) \sim (9)\; \\ \end{gathered} \right. \hfill \\ \end{gathered}$$

The multi-objective particle swarm optimization algorithm(MOPSO) is an effective optimization tool for nonlinear optimization problems, combinatorial optimization problems and mixed integer nonlinear optimization problems. The algorithm is concise, easy to implement, does not require many parameters to be adjusted, and does not require gradient information, making it particularly suitable for engineering applications. Therefore, MOPSO is adopted in the paper. The particle swarm optimization is prone to falling into local optima. So it is necessary to increase the inertia weight and dynamic weighting method to select guiding particles, ensuring that the optimization results avoid falling into local optima. The mutation operation can enhance the global search ability of particles and improve the diversity of solutions.

In the search space of dimension $n$, there are $m$ particles forming the population, where the position of the $i$ particle is $x_{i} = \left\{ {x_{i1} ,x_{i2} ,...,x_{im} } \right\}$ and its velocity is $v_{i} = \left\{ {v_{i1} ,v_{i2} ,...,v_{in} } \right\}$. Among them, the individual extreme value is $p_{i} = \left\{ {p_{i1} ,p_{i2} ,...,p_{in} } \right\}$, the optimal value of the population is $p_{g} = \left\{ {p_{g1} ,p_{g2} ,...,p_{gn} } \right\}$, and the particle $x_{i}$ updates the velocity and position of each dimension as follows:

32 $$\begin{aligned} v_{id}^{z + 1} & = wv_{id}^{z} + c_{1} r_{1} \left( {p_{id}^{z} - x_{id}^{z} } \right) + c_{2} r_{2} \left( {p_{gd} - x_{gd} } \right) \\ x_{id}^{t + 1} & = x_{id}^{t} + v_{id}^{t + 1} \\ \end{aligned}$$

where, $$d = 1,2,...,n,i = 1,2,...,m,$$$$m$$ is the population size. $$z$$ is the current evolutionary algebra. $$r_{1} ,r_{2}$$ are random numbers distributed between $$\left[ {0,1} \right]$$ and independent of each other.$$c_{1} ,c_{2}$$ are acceleration factors. $$w$$ is adapted according to Eq. (32) to enhance the algorithm's global search ability. Adaptive $$c_{1}$$ and $$c_{2}$$ are adopted in this paper. In the initial stage, the local search ability is enhanced with a larger $$c_{1}$$, and in the later stage, the global search ability is enhanced with a larger $$c_{2}$$. $$c_{1}$$ and $$c_{2}$$ are determined by Eqs. (34) and (35) respectively:

33 $$w = w_{0} + r\left( {w_{1} - w_{0} } \right)$$

34 $$c_{1} = \left( {c_{1f} - c_{1i} } \right)\frac{z}{Z} + c_{1i}$$

35 $$c_{2} = \left( {c_{2f} - c_{2i} } \right)\frac{z}{Z} + c_{2i}$$

where, $w_{0}$ is between $\left[ {0,1} \right]$, $w_{1} - w_{0} > 0$. It is recommended $w_{0}$ to be $\left[ {0,0.5} \right]$, $r$ is a random number of distribution of $\left[ {0,1} \right]$, $c_{2f}$, $c_{2i}$, $c_{1i}$, $c_{1f}$ are all constants and $Z$ represents the total number of iterations.

In the previous work, a dynamic weighting method is proposed to select guiding particles. In multi-objective optimization problems, the optimal particle is the optimal frontier, which is a set. Press Eq. (36) to select the guiding particles. Calculate the fitness of each particle in the solution set with Eq. (36). The particle with the highest current fitness is the globally optimal particle. In the equation, $w_{i}$ is a random value, and $M$ is the optimal number of particles.

36 $$\begin{aligned} & ff = 1/\sum\limits_{i = 1}^{M} {w_{i} f\left( x \right)_{i} } \\ & \sum\limits_{i = 1}^{M} {w_{i} = 1} \\ \end{aligned}$$

Mutation operations can enhance the global search ability of particles and improve the diversity of solutions. When a mutation produces an excellent particle, it can attract other solutions, thereby helping to escape from local optima. Therefore, MOPSO adopts a mutation strategy to enhance the escape ability of particles towards local optima.

The mutation strategy adopted by MOPSO is that when the flight speed of the entire group of particles is less than a limited value, the velocities of certain particle on certain dimensions are randomly changed within the specified range to increase the global search ability of the particles. The specific mutation operation is shown in Eqs. (37) and (38).

37 $$v_{m} = 2\left( {r_{3} - 1} \right)\Upsilon \varpi_{m}$$

38 $$x_{id}^{t + 1} = x_{id}^{t} + v_{m}$$

where, $v_{m}$ is the value of variation, $\Upsilon$ is the coefficient of variation of [0,1], which is used to regulate the degree of variation, $r_{3}$ is a random number that varies within the range of [0, 1], $x_{id}^{t}$ represents the randomly selected dimension $d$ of the particle $i$.

**Figure 2:**
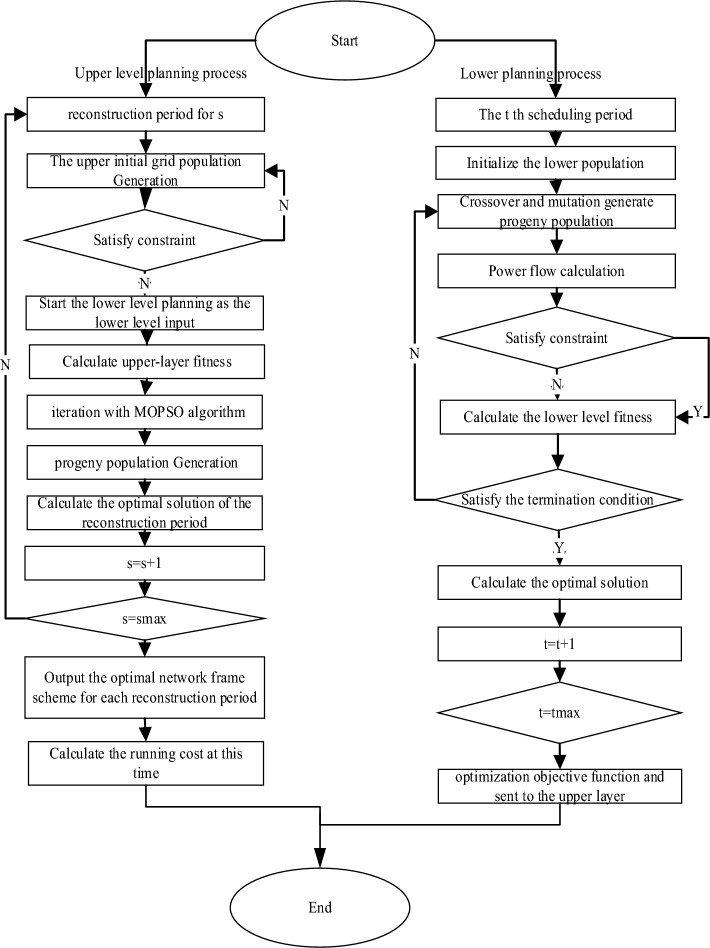
The flow chart of optimization algorithm (figure shows the logic block diagram of two-level optimization solution.)

At present, MOPSO has been widely used in function optimization, neural network training, fuzzy system control and other application fields of genetic algorithms^26,27^. The advantage of MOPSO is that the algorithm is simple and easy to implement, there are not many parameters to adjust, and no gradient information is required, which is especially suitable for engineering applications. Therefore, multi-objective optimization algorithm based on particle swarm optimization algorithm is adopted in this paper. The parameters are set as follows: the initial population number is 50, the maximum number of iterations is 50, the crossover rate is 0.9, and the variation rate is 0.1. The switch action cost is 5 yuan/time, the network loss cost is 0.5 yuan /kWh, and the steel price is subject to the actual industry benchmark price, which is 2500 yuan/ton in this paper. The node power distribution system is in the attchment. Taking an steel plant park in Northeast China as an example,the load mainly includes 9 steel production plants. In these nine plants, power nodes are defined as 1, 3, 6, 8, 9, and steel nodes are defined as 4, 7, 11, 5, 2, 14, 10, 12, 13. The branch impedance and related parameters of the system are shown in S1. The branch parameters are shown in S2. The real-time electricity price and demand response price are shown in S3.The load classification of steel plants is shown in S4. The number and power of rolling mills in steel plants is shown in S5. The number and power of electric arc furnaces are shown in S6. Pumps, blowers, dust removal machines, conveyors and other loads are regarded as constant power loads, electric arc furnaces, rolling mills as impact loads. The model in this paper is used to identify parameters with production data. The optimization solution process is shown in Fig 2.

The uncertainty on the load side is solved as follow:

In terms of load uncertainty, the uncertainty in this paper mainly comes from the uncertainty of electricity consumption on the load side. There is a significant deviation between the predicted value and the actual value caused by subjective or objective reasons. The situation is solved by cluster based on the historical load of consumer in the park according to the literature^28^. Thus, the uncertainty confidence level of the load and the power fluctuation caused by uncertainty can be obtained. Day-ahead optimization is the main optimization time scale of this paper. The deviation can be reduced by correcting it with intraday optimization. By using the BP neural network algorithm to learn, the power fluctuation can minimize the deviation caused by uncertainty as much as possible. Translate uncertainty into predictive values learned from historical data to solve uncertainty problems. The uncertainty state confidence level is defined as the ratio of the difference between the theoretical adjustable ability and the actual ability at a certain moment to the theoretical data, specifically:

39 $$R\left( t \right) = \frac{{a_{k}^{\prime } \left( t \right) - a_{k} \left( t \right)}}{{a_{k}^{\prime } \left( t \right)}}$$

where, $R\left( t \right)$ is the confidence level, $A^{\prime } \left( t \right)$ is the theoretical data at time t, and $A\left( t \right)$ is the actual data at time *t*. The confidence level will affect the load clustering results.

For the example, the following table is obtained by learning the deviation values. The deviation values for a certain day are shown in Table 1. From this table, it can be seen that the maximum deviation value through learning is 10%. In actual production and life, there are many uncertain factors, and this is also the focus of the authors next research, how to calculate the carbon emissions , energy costs and optimize schedul of load based on various uncertainties.

**Table 1:** Deviation value caused by load uncertainty.

Time/h	0	1	2	3	4	5	6	7	8	9	10	11
Forecast bias	0.06	0.10	0.09	0.10	0.07	0.09	0.10	0.08	0.10	0.08	0.09	0.10
Time/h	12	13	14	15	16	17	18	19	20	21	22	23
Forecast bias	0.04	0.05	0.09	0.08	0.08	0.07	0.10	0.08	0.09	0.02	0.01	0.09

## Discussion

(1) The universality and parameter sensitivity of the model.

**Figure 3:**
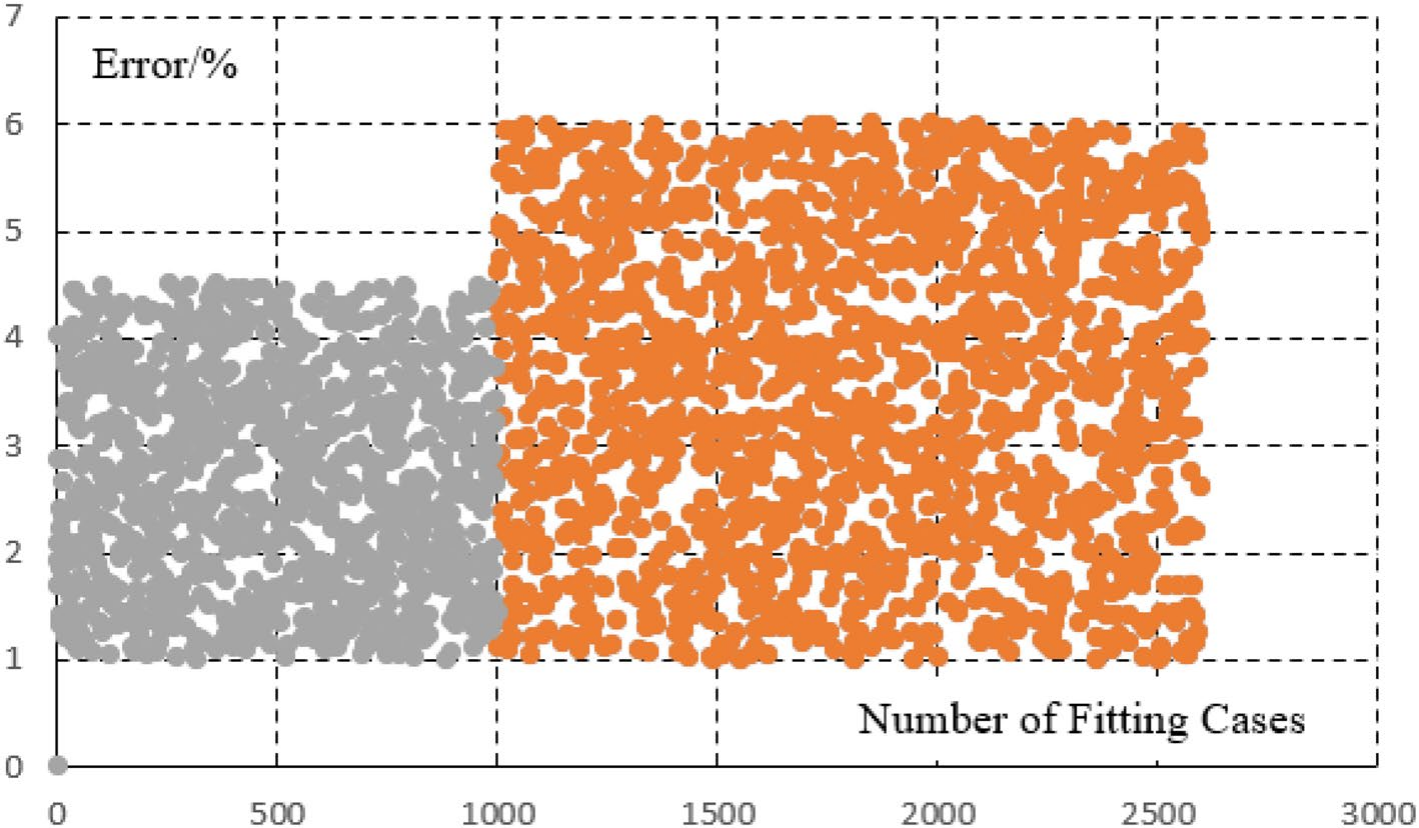
Error analysis of large scale stable load.

**Figure 4:**
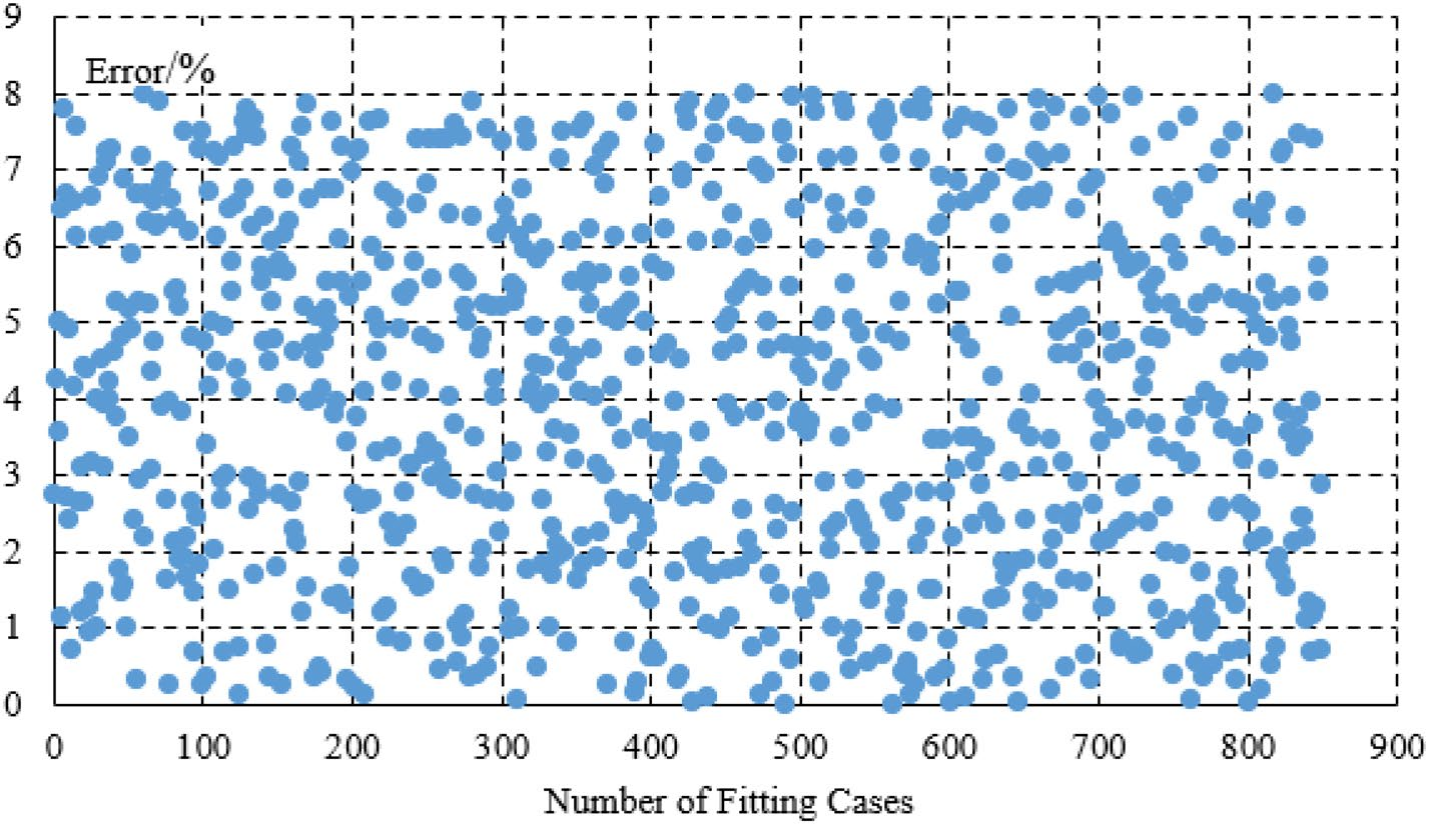
The fitting diagram of the impact load.

**Figure 5:**
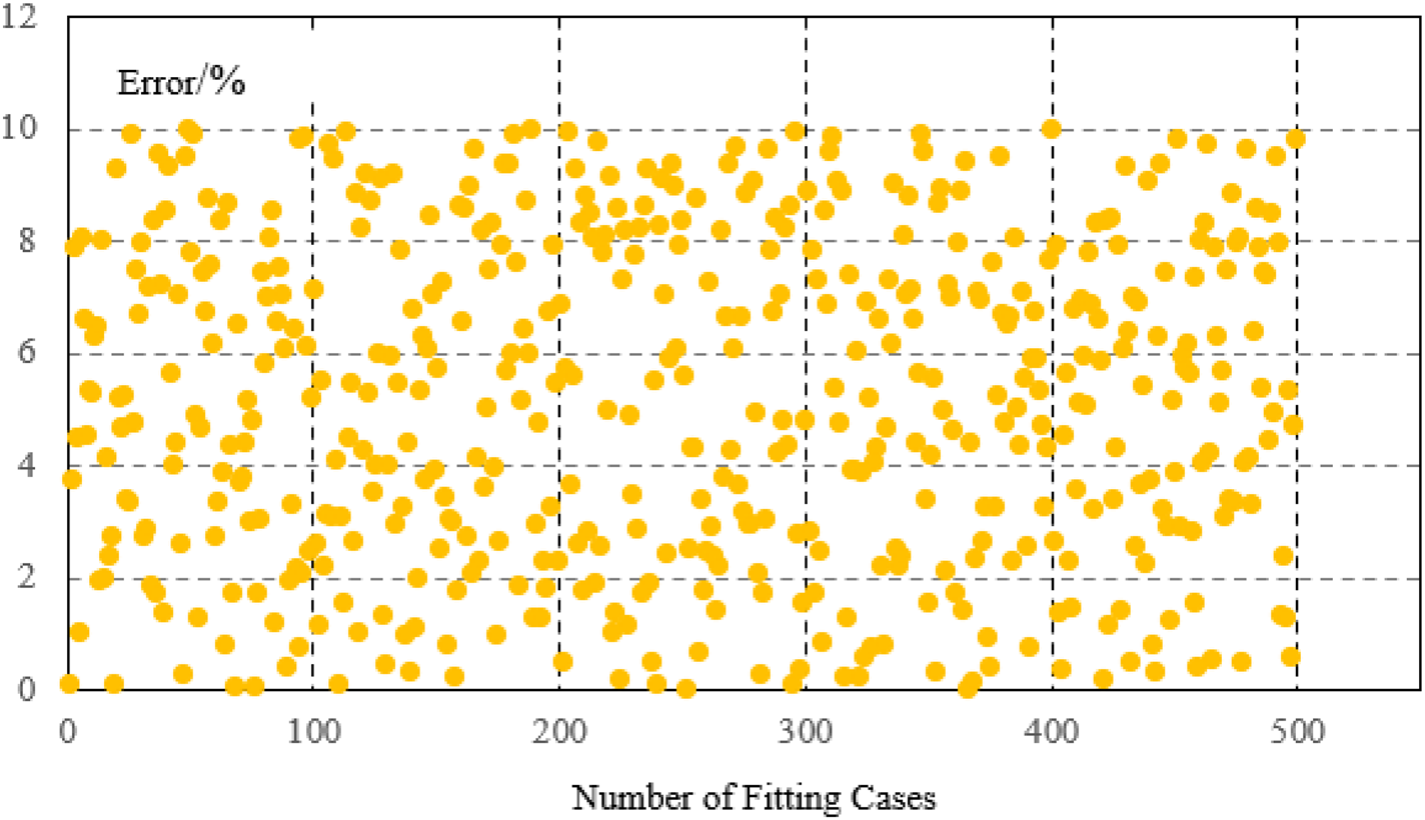
The fitting error of the load production line.

The steel plants are generally large in scale and have a large number of equipments. In this paper, the load is divided into static load, impact load, and production line load.The burden of model establishment is reduced through load classification. The calculation example in the paper is a large-scale steel plant park in Northeast China. However, prior to this emample, the model had already undergone extensive parameter identification and accuracy comparison of the model. Stable load generally has small power fluctuations and is not easily affected by external factors. The electricity consumption behavior is stable. By fitting the parameters and operating power of 2300 stable loads in steel plants, the calculation error is within 6%. From this error analysis, it can be seen that there are 1000 continuous operation submerged arc furnaces (with high power but stable load for a long time), which have relatively small fluctuation characteristics It can generally be maintained within 4.5%. There are 1300 conveyors, water pumps, etc. (with frequent fluctuations in load but low power), and these loads also meet an error of within 6%. However, due to their small power, the impact of this power error is not significant, as shown in Fig. 3. For impact loads, the average error of fitting 850 impact loads is 8%, and the maximum error of fitting 500 production line loads is 10%, as shown in Figs. 4 and 5.

From the mathematical perspective, the sensitivity of a design function $F_{j} \left( X \right)$ to the design variable $X_{i}$ at a certain design point $X_{k}$.

where, $\left| {S_{{{\text{ji}}}} } \right|$ is the sensitivity of the function $F_{j} \left( X \right)$ to $X_{i}$. The larger the value, the greater the sensitivity and the greater the impact on the numerical model.

**Figure 6:**
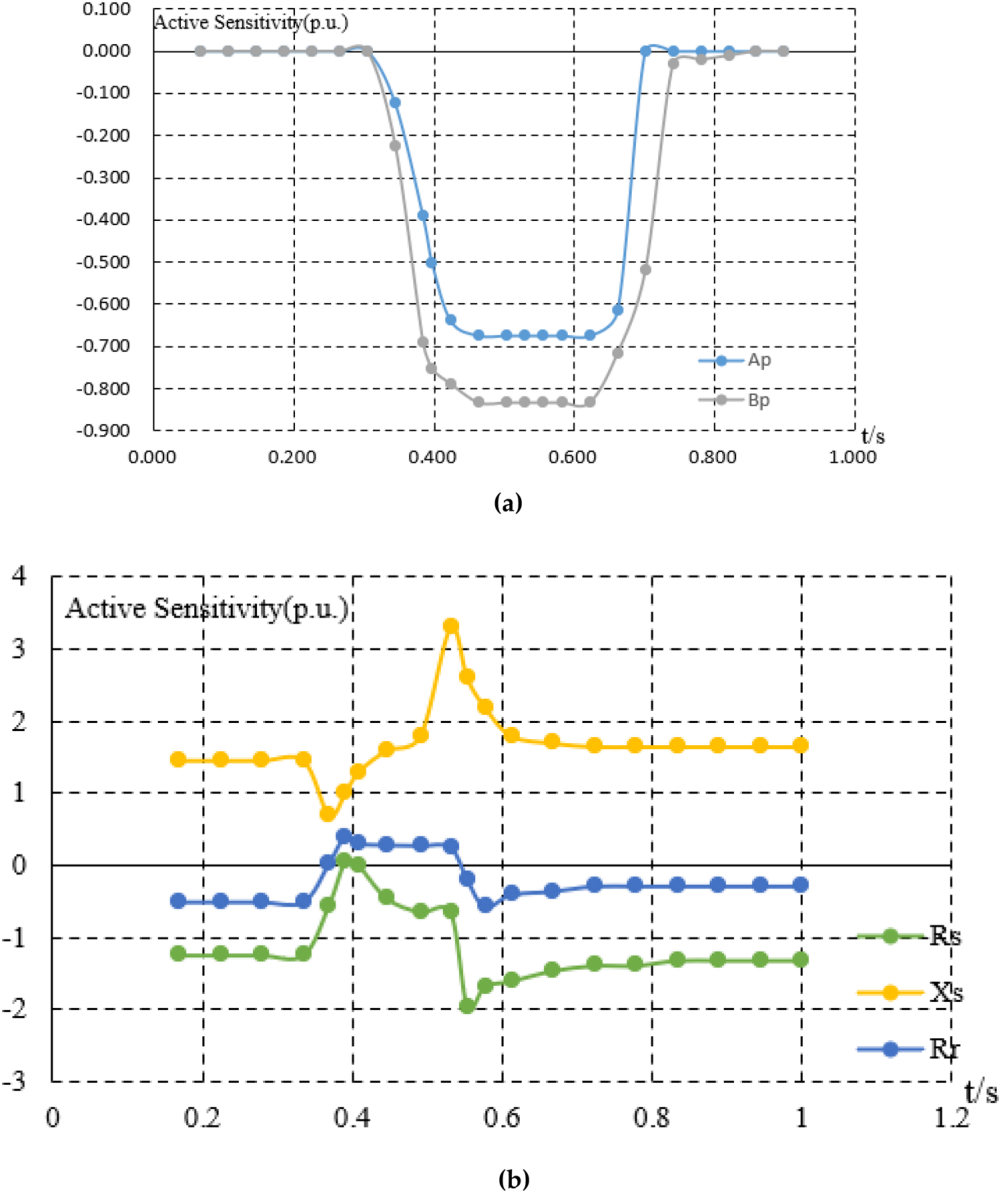
(**a**) The sensitivity analysis of the static load. (**b**) The sensitivity analysis of the impact load.

The sensitivity of the load is shown in the Fig. 6.

ZDT1–ZDT4 is adopted to verify the performance of MOPSO. The convergence Υ And diversity Δ is adopted evaluate the convergence and diversity of algorithms. The algorithms NSGA-II and PESA-II, NSPSO, RM-MEDA is adopted to compare with MOPSO. The population size of NSGA-II and NSPSO is 100, with 250 iterations.The PESA-II population size is 10 and the number of iterations is 2500. Function evaluations are all 25,000 times. The output Pareto solution sets are all 100 in size. Take 0.9 for pc in NSGA-II and PESA-II, with pm = 1/n. In NSPSO, c1 = 1, c2 = 2, and w decrease linearly from 1.0 to 0.4. The Pareto solutions of the MOPSO algorithm on ZDT1–ZDT4 are shown in Tables 2 and 3.

**Table 2:** The comparison of convergence Υ (M-mean, VAR variance).

	ZDT1	ZDT2	ZDT3	ZDT4
NSGA-II				
(M)	0.033	0.072	0.0111	0.051
(VAR)	0.002	0.028	0.006	0.011
PESA-II				
(M)	0.001	0.008	0.006	9.96
(VAR)	0.000	0.007	0.000	18.88
NSPSO				
(M)	0.005	0.007	0.002	6.26
(VAR)	0.000	0.000	0.004	6.95
RM-MEDA				
(M)	0.022	0.027	0.042	50.68
(VAR)	0.000	0.000	0.006	5.39
MOPSO				
(M)	0.000	0.002	0.00	0.16
(VAR)	0.000	0.000	0.000	0.001

**Table 3:** The comparison of diversity Δ (M-mean, VAR variance).

	ZDT1	ZDT2	ZDT3	ZDT4
NSGA-II				
(M)	0.39	0.42	0.75	0.7
(VAR)	0.002	0.003	0.015	0.05
PESA-II				
(M)	0.781	0.862	1.206	0.998
(VAR)	0.002	0.006	0.025	0.000
NSPSO				
(M)	0.885	0.912	0.601	0.997
(VAR)	0.923	0.966	0.001	0.001
RM-MEDA				
(M)	0.385	0.356	0.822	0.787
(VAR)	0.000	0.004	0.003	0.005
MOPSO				
(M)	0.566	0.533	0.485	0.446
(VAR)	0.001	0.000	0.000	0.001

(2).The analysis of optimization results

**Figure 7:**
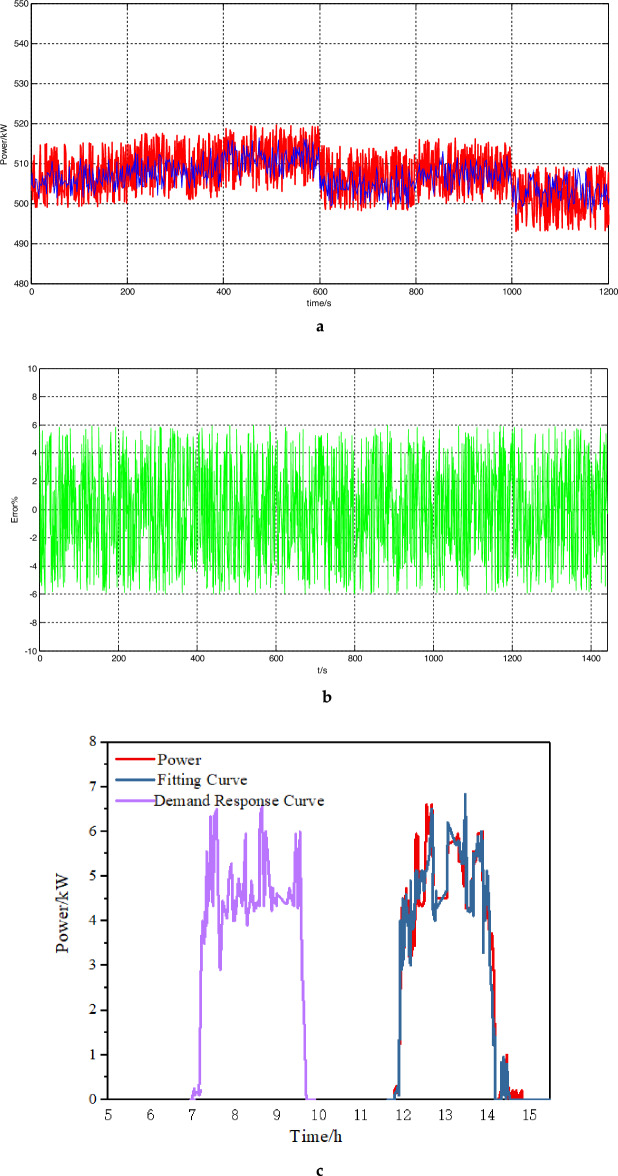

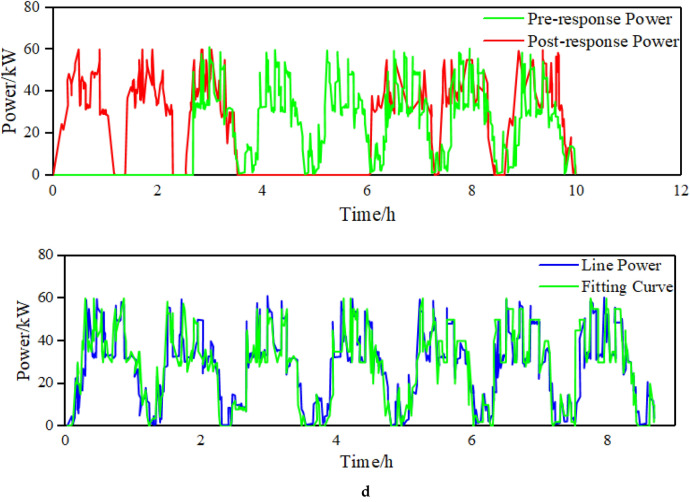
(**a**) The static load fitting curve (figure is the fitting results of the stable power loads in the park. a is the simulation error diagram of stable load). (**b**) The curve of the static load simulation error. (**c**) The load curve of the impact load. (**d**) The load curve of the production line

Figure 7 is the fitting results of the stable power loads in the park. Figure 7a is the simulation error diagram of stable load. As shown in the Fig. 7b, the load model can better fit the actual power of the load, which provides a good basis for improving energy efficiency on the load side and accurately calculating the power of the load participating in the demand response. For the stable load, the calculation error on the load side can be seen to be within 6%. The average error of imppact load fitting is 8%, The maximum error of production line load fitting is 10%, Which is within the allowable error range of 20% of the actual implementation error of the demand response project in China.

It shows the simulation diagram of impact load and the comparison diagram before and after participating in demand response of the steel plant in node 11 in Fig. 7c. The load model can reflect the properties of the load and conform to the allowable error range. In the context of energy efficiency optimization, the impact load changes the production time without affecting the total production, arranges production at a time when the electricity price is low and the demand response is high.It reduces the energy consumption cost, and alleviates excess energy. As can be seen in Fig. 7d, production line load participates in demand response without affecting production. In a single scheduling cycle, by changing the production running time to participate in the consumption at night and peak cutting during the day, the load energy planning is improved.

**Figure 8:**
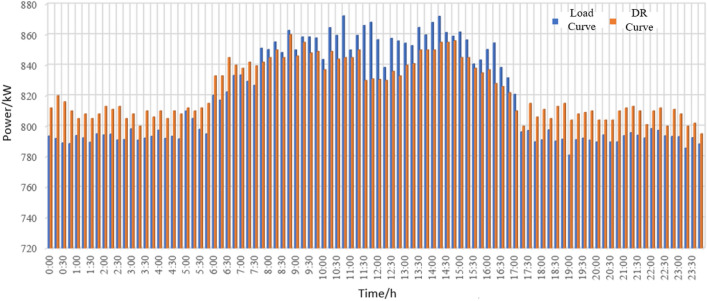
Load characteristic curve and demand response curve.

From Fig. 8, it can be seen that due to the optimization of energy efficiency on the load side, it has a certain demand response capacity, providing a certain space for power flow and grid security and stability. Participating in demand response at night to increase electricity consumption can reduce nighttime energy losses and improve the efficiency of line energy transmission. The demand response during the day can not only improve the energy efficiency on the load side, but also reduce the line load, avoid the peak valley difference caused by the peak electricity consumption during the day.It also enhance the stability of the power grid and emergency adjustable capacity. Therefore, in the steel plants, electric arc furnaces and steel rolling are the main demand response objects.

The power grid reconstruction can slow down network energy loss. However, the mechanical losses and reconstruction costs caused by frequent reconstruction are generally not economical. There are significant differences in load electricity consumption among the four time periods. According to the division method in reference^29^, typical days are divided into four reconstruction time periods, namely 1:00–6:00, 7:00–10:00, 11:00–20:00, and 21:00–24:00. At the beginning of each reconstruction period, the network structure undergoes reconstruction, while during one reconstruction period, the network struc-ture remains unchanged.

From the attachment, it can be seen that the only one branch needs to be disconnected from each basic closed loop, and the common branch part of adjacent basic closed loops can only disconnect one branch at most. The grid population generated according to the above rules can ensure that the radial constraint conditions are met, making the upper level optimization become unconstrained optimization.

The network is reconstructed at the beginning of each reconstruction period, and the net-work remains unchanged during one reconstruction period. The grid simulation results of the proposed method are shown in Table 4. The line has only two states of access and disconnect, which are 0–1 variables, so the conventional grid variables are encod-ed based on binary. Therefore, for an system with 14nodes, the grid must have 13 branches. At the same time, it is also necessary to meet the requirements of no islands and no loops in the grid. As can be seen from the attachment, the system with 14nodes has four basic closed loops, so only one branch of each basic closed loop needs to be disconnected, and the total branches of adjacent basic closed loops can only be disconnected at most one branch. The grid species group generated according to the above rules can ensure that the radiation constraint conditions are satisfied, and the upper optimization becomes unconstrained optimization. When considering load network reconstruction, due to changes in the system network structure and different network flows, losses will also change accordingly, resulting in different overall costs. When switching, there will be a certain reconstruction cost.

**Table 4:** Optimization comparison table.

Time	Disconnect branch	Transmission loss before/kW	Transmission loss after/kW
0: 00–6: 00	a, d, m, j, s, p, t	12.834	7.662
6: 00–13: 00	b, e, f, k, n, o, n	65.284	43.082
13: 00–19: 00	a, f, m, i, j, p, n	102.13	84.45
19: 00–24: 00	b, h, i, j, s, o, t	45.78	31.11

**Figure 9:**
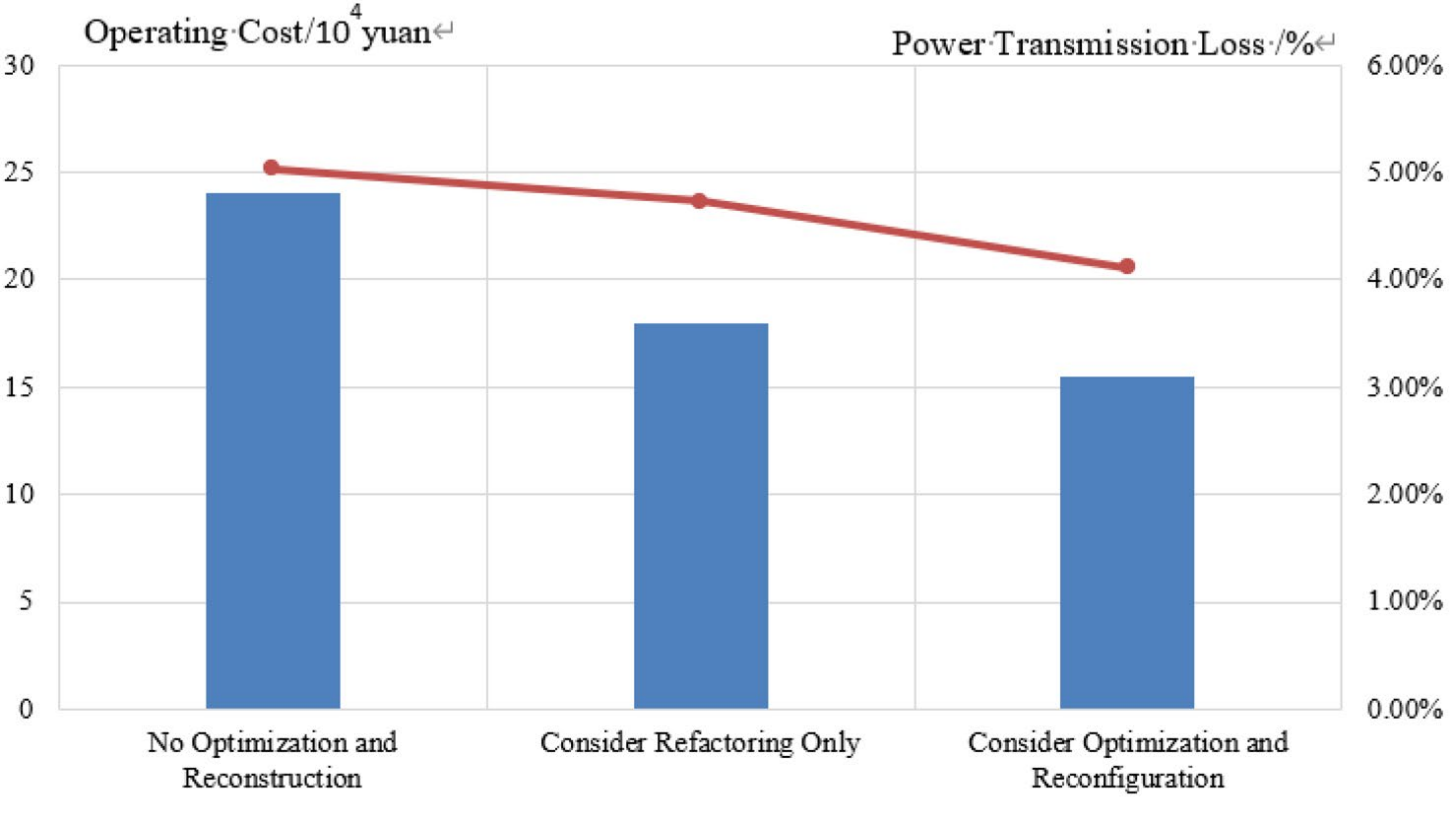
Comparison of network loss and operating costs in various scenarios

This paper demonstrates the unique advantages of this method by comparing several different scenarios. Considering the same data as above, consider the following scenarios:

(1) Regardless of load side energy efficiency optimization and distribution network optimization. (2) Consider load side energy efficiency optimization, but do not consider distribution network optimization. (3) Not considering load side energy efficiency op-timization, but considering distribution network optimization. (4) Considering both load side energy efficiency optimization and distribution network optimization. In scenario 1), the system has the highest operating cost. At this time, the load operation only relies on production scheduling, without considering energy efficiency improvement or network loss. Therefore, the network loss is also the highest at 10.8%, and the energy cost is 125.08 × 10^4^ yuan. The overall operation is not economical, resulting in increased costs and waste of electricity. The production process and time should be reasonably arranged while ensuring load production, and the advantages of continuous and adjustable electric arc furnace and translational steel rolling production line should be fully utilized without delaying production operation. In scenario 2), due to the consideration of power grid reconstruction, the system reconstruction cost is increased by 3.41 × 10^4^ yuan, but at this time, the energy loss between power grid transmission decreased by 1.2%, and the economic efficiency of power grid transmission increased. In scenario 3), due to the consideration of load side optimization, the load energy consumption time has been changed, and the participating demand response capacity has been increased. Due to a decrease in daytime energy consumption and an increase in nighttime energy consumption, the overload situation of the lines during the day have been indirectly improved, while nighttime electricity consumption has increased, indirectly reducing line loss. When the load on the transmission line is relatively low, the fluctuation on the load side has a significant impact on the power loss of the transmission line. The comparison diagram of network loss and operating cost for each scenario is shown in Fig. 9.

In scenario 4), it can be seen from Table 5 that the total operating cost when considering reconstruction and load side electricity cost optimization is 1.0285 million yuan, while the total operating cost when not considering optimization is 125.08 × 10^4^ yuan, reducing the annual operating cost by 17.77%. The line loss has decreased by 1.8%. However, due to system reconstruction and rearrangement of transmission capacity, although a reconstruction cost of 3.41 × 10^4^ yuan may be incurred, the overall cost has decreased while reducing the load pressure on some heavy-duty lines, reducing the overall operating cost of the power grid while reducing static line loss and reducing the burden on the power grid. In senario 4), compared to the first three scenarios, both from the grid side and the load side, the economy and network losses are the best. The cost savings and demand response income are shown in Table 6, and the comparison between electricity loss and operating costs under each scenario is shown in Table 5.

**Table 5:** Comparison of energy consumption costs before and after optimization.

Optimization objective	No optimization and reconstruction	Consider optimization only	Consider optimization and Reconfiguration
Reconstruction cost/10^4^ yuan	0	0	3.41
Energy cost/10^4^ yuan	125.08	102.85	102.85

**Table 6:** Comparison of Energy Cost Savings and Demand Response Revenue.

Optimization objective	Consider optimization and reconfiguration	No optimization and reconstruction	Consider refactoring only	Consider optimization only
Demand response benefits	6.65	0	0	6.65
Electricity cost savings	15.58	0	0	15.58
Cost savings in grid operation	10	0	7.94	0

**Figure 10:**
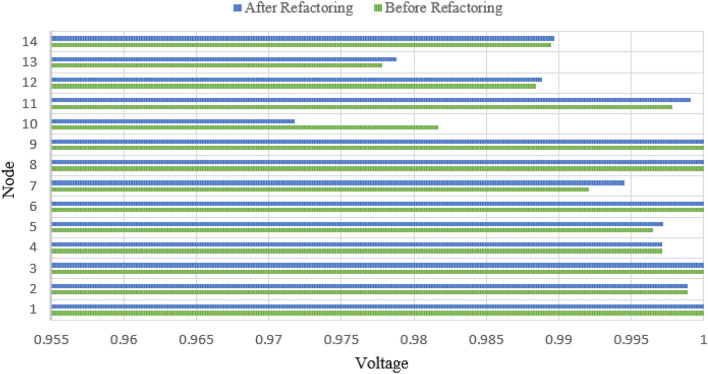
The comparison table of voltage before and after.

The overall voltage after reconstruction is closer to the rated voltage than before. Due to the power grid reconstruction, the power flow is more uniform, avoiding the problem of low terminal voltage caused by line overload. The comparison table of voltage before and after is shown in Fig. 10. There are also varying degrees of improvement in voltage before and after optimization, with a 1.0% improvement in voltage for node 10.

## Conclusion and outlook

In this paper, an optimization algorithm for the energy efficiency of the whole network considering dynamic network reconstruction and static network loss is proposed. According to the simulation results, the following conclusions are obtained:

1. The mathematical models of steel stable load, impact load, and production line load based on the electrical properties and time-domain characteristics is built in the paper. The results show that calculation error of the stable load can be arranged within 6%, and the allowable range of error between the impact load and the production line load is within 8%.
2. A load energy efficiency optimization model is proposed based on the definition of energy efficiency ratio. By comparing four different scenarios, it can be concluded that the total operating cost when considering reconstruction and load side electricity cost optimization is 1.0285 million yuan, while the total operating cost when not considering optimization is 1.2508 million yuan, reducing the annual operating cost by 17.77%. The line loss has been decreased by 1.8%. There are also varying degrees of improvement in voltage before and after optimization, with a 1.0% improvement in voltage for node 10.

In terms of modeling, the errors are all within the allowable range, which is in line with the allowable error range of 20% in response to the actual engineering implementation error in China's demand response.In terms of optimization, by further calculating the cost of static network loss and dynamic network reconstruction, the energy efficiency of the power grid and load side can be improved, and the economy of energy transmission and energy load can be improved Figs. 4, 5, 6.

For steel plants, there are various forms of load and types of energy demand. It only sonsiders the load energy efficiency and operating costs involved in grid regulation in steel plants in the paper. How to further reduce the comprehensive energy consumption cost of the load and reduce the electricity consumption cost including carbon emission costs remains a problem that needs to be studied. How to accurately calculate load side carbon emissions and optimize energy utilization scheduling based on various uncertainties still needs to be considered in detail (Tables 1, 2, 3).

### Supplementary Information

Supplementary Information.

## Data Availability

The data that supportthe findingsof this study are available from the corresponding author upon reasonable request.
