# Supplementary material for: Comprehensive energy efficiency optimization algorithm for steel load considering network reconstruction and demand response
Source: Sci Rep. 2023 Nov 21;13:20345. doi: 10.1038/s41598-023-46804-7 (PMC10663541; doi:10.1038/s41598-023-46804-7)
Supplement: Supplementary file 1 — Supplementary Information. [file 41598_2023_46804_MOESM1_ESM.docx]

**S1** Steel plant park system

| Branch | Branch resistance | Branch reactance | 1/2 capacitance susceptance |
| --- | --- | --- | --- |
| a | 0.01938 | 0.05917 | 0.02640 |
| b | 0.04699 | 0.01979 | 0.02190 |
| c | 0.05811 | 0.17632 | 0.01870 |
| d | 0.05403 | 0.22304 | 0.02460 |
| e | 0.05695 | 0.17388 | 0.01700 |
| f | 0.06701 | 0.17103 | 0.01730 |
| g | 0.01335 | 0.04211 | 0.00640 |
| h | 0.0000 | 0.17615 | 0.00000 |
| i | 0.0000 | 0.11001 | 0.00000 |
| j | 0.03181 | 0.08450 | 0.00000 |
| k | 0.09498 | 0.19890 | 0.00000 |
| l | 0.12291 | 0.15581 | 0.00000 |
| m | 0.06615 | 0.13027 | 0.00000 |
| n | 0.12711 | 0.27038 | 0.00000 |
| o | 0.08205 | 0.19207 | 0.00000 |
| p | 0.22092 | 0.19988 | 0.00000 |
| q | 0.17093 | 0.34802 | 0.00000 |
| r | 0.0000 | 0.25202 | 0.00000 |
| s | 0.0000 | 0.20912 | 0.00000 |
| t | 0.0000 | 0.55618 | 0.00000 |

**S2** Parameters of branch

| Time | Real-Time price  (yuan·kWh^-1^) | demand response price |
| --- | --- | --- |
| 10:00-15:00;  17:00-22:00 | 1.2 | 0.35 |
| 6:00-10:00;  15:00-17:00  22:00-24:00 | 0.8 | 0.2 |
| 00:00-6:00 | 0.35 | 0.1 |

**S3** Real-Time price and demand response price

|  | Load Category | Load name |
| --- | --- | --- |
| Steel | static model | Oxygen generator, water pump, conveyor, blower |
|  | Impact model | Rolling mill, electric arc furnace |

**S4** Load facility classification

|  | Rough-rolled steel | Power  (kW) | Fine rolled steel | power (kW) | Gross power | Gross capacity |
| --- | --- | --- | --- | --- | --- | --- |
| 4 | 2 | 100 | 5 | 55 | 475 | 800 |
| 7 | 1 | 115 | 5 | 50 | 365 | 600 |
| 11 | 2 | 100 | 4 | 60 | 540 | 850 |
| 5 | 2 | 850 | 6 | 100 | 2300 | 3200 |
| 2 | 1 | 100 | 7 | 70 | 590 | 1800 |
| 14 | 3 | 130 | 8 | 110 | 1270 | 1900 |
| 10 | 3 | 155 | 3 | 110 | 795 | 2000 |
| 12 | 5 | 130 | 4 | 90 | 810 | 2200 |
| 13 | 4 | 100 | 3 | 90 | 670 | 1500 |

**S5** The number and power of rolling mills in steel plants

| Electric arc furnace | Power |
| --- | --- |
| 4 | 200 |
| 7 | 150 |
| 11 | 200 |
| 5 | 250 |
| 2 | 500 |
| 14 | 200 |
| 10 | 400 |
| 12 | 300 |
| 13 | 250 |
| 4 |  |

**S6** Number and power of electric arc furnaces
